# Systematic Analysis of the *Betula platyphylla TCP* Gene Family and Its Expression Profile Identifies Potential Key Candidate Genes Involved in Abiotic Stress Responses

**DOI:** 10.3390/plants14060880

**Published:** 2025-03-11

**Authors:** Shengzhou Guo, Yuan Xu, Yi Zhou, Ronglin Liu, Yongkang Wang, Ling Yao, Syed Muhammad Azam, Huanhuan Ma, Xiaomin Liu, Shijiang Cao, Kang Wang

**Affiliations:** 1 College of Forestry, Fujian Agriculture and Forestry University, Fuzhou 350002, China; gsz19559162600@126.com (S.G.); 13792766239@163.com (Y.X.); d1315068727@163.com (R.L.); wykkk1890@126.com (Y.W.); 2 College of Forestry, Zhejiang Agriculture and Forestry University, Hangzhou 311300, China; 13353338512@139.com; 3 Fujian Provincial Key Laboratory of Soil Environmental Health and Regulation, College of Resources and Environment, Fujian Agriculture and Forestry University, Fuzhou 350002, China; yl1743024270@163.com; 4 Institute of Environmental Microbiology, College of Resources and Environment, Fujian Agriculture and Forestry University, Fuzhou 350002, China; syedazamfafu@gmail.com; 5 State Key Laboratory of Tree Genetics and Breeding, College of Biological Sciences and Technology, Beijing Forestry University, Beijing 100083, China; mhh00928@163.com (H.M.); liuxiaomin@bjfu.edu.cn (X.L.)

**Keywords:** TCP transcription factor, *Betula platyphylla*, abiotic stress, subcellular localization, qRT-PCR expression profiling

## Abstract

The TCP transcription factor (TF) family is a vital set of plant-specific regulators involved in plant growth, development, and responses to environmental stresses. Despite the extensive research on TCP transcription factors in numerous plant species, the functions they fulfill in *Betula platyphylla* are still not well understood. In this study, 21 *BpTCP* genes were identified via genome-wide analysis. Bioinformatics analysis was used to examine the physicochemical properties of these transcription factors, including molecular weight, isoelectric point, chromosomal distribution, and predicted subcellular localization. We expected that most BpTCP transcription factors would be located in the nucleus. Collinearity analysis revealed that gene fragment duplication events played a major role in the evolutionary expansion and diversification of the *BpTCP* gene family. Promoter analysis identified diverse *cis*-acting elements in *BpTCP*, suggesting that they play a role in stress responses, hormonal regulation, and plant growth and development. qRT-PCR analysis showed that *BpTCP* genes displayed tissue-specific expression patterns in the roots, stems, and leaves, displaying remarkable differences in expression levels when subjected to abiotic stresses, including drought and high- and low-temperature conditions. Notably, *BpTCP17* and *BpTCP18* showed markedly higher expression levels under multiple stress conditions. Subcellular localization experiments confirmed that both *BpTCP17* and *BpTCP18* localize in the nucleus, consistent with bioinformatic predictions. These findings emphasize the potential roles of *BpTCP17* and *BpTCP18* in mediating abiotic stress responses, highlighting their potential as candidate genes for improving stress tolerance in *B. platyphylla*.

## 1. Introduction

Plants, as sessile organisms, depend on complex transcriptional regulatory mechanisms to adapt to environmental stresses, in which transcription factor (TF) networks play a vital role [1]. The *TCP* (TEOSINTE BRANCHED1, CYCLOIDEA, PROLIFERATING CELL FACTORS1/2) gene family is a plant-specific TF family that regulates responses to hormones, nutrients, and environmental stresses while contributing to cell proliferation and organ development [2,3]. Named after founding members [4] in maize (TB1) [5], snapdragon (CYC) [6], and rice (PCF1/2) [7], TCP transcription factors possess a conserved basic helix–loop–helix (bHLH) domain essential for DNA binding and protein interactions [8]. This domain features unique structural characteristics that enable distinct DNA-binding properties [9]. TCP transcription factors are categorized into two subgroups: class I (TCP-P/PCF) and class II (TCP-C). Class I TCPs, such as PCF1/2 in rice, have shorter basic regions, whereas class II TCPs are further divided into CIN and CYC/TB1 subclasses [10]. CIN genes, like TCP2, TCP3, and TCP4 in *Arabidopsis thaliana*, are regulated by miR319 [11], while CYC/TB1 genes are specific to angiosperms and include an ECE domain associated with protein interactions [12]. With the advent of genome sequencing across diverse plant species and advances in bioinformatics, the *TCP* gene family has been extensively identified and analyzed in various plants. Examples include tomato (*Solanum lycopersicum*) [13], switchgrass (*Panicum virgatum*) [14], kiwifruit (*Actinidia deliciosa*) [15], chili pepper (*Capsicum annuum*) [16], maize (*Zea mays*) [17], soybean (*Glycine max*) [18], and petunia (*Petunia* spp.) [19]. These studies provide a rich foundation for understanding the roles of *TCP* genes in plant development and adaptation to environmental adversities, offering critical insights into their functional diversification and regulatory mechanisms.

The pivotal roles of TCP transcription factors in plant growth and development have gained widespread recognition. These transcription factors regulate a broad spectrum of essential biological processes, including seed germination [20,21], branch formation [22], floral organ morphogenesis [23,24], and leaf growth [25]. In *Arabidopsis thaliana*, *TCP* genes such as *TCP2*, *TCP3*, *TCP4*, and *TCP10* orchestrate leaf development by modulating the central and marginal regions through the regulation of *miR319* expression [26]. Similarly, in tomato (*Solanum lycopersicum*), SlTCP proteins have been implicated in growth regulation via heterodimer formation [13]. In cucumber (*Cucumis sativus*), the *TCP* family gene *CsBRC1* modulates lateral bud growth by directly repressing *CsPIN3* expression [27]. In banana (*Musa acuminata*), *MaPCF10* and *MaPCF13* have been linked to fruit development and ripening [28]. In potato (*Solanum tuberosum*), *StTCP15* mediates tuber emergence by maintaining a dynamic balance between abscisic acid (ABA) and gibberellins [29]. While substantial research has illuminated the regulatory functions of TCP transcription factors in plant growth, development, and morphogenesis, emerging evidence emphasizes their critical roles in the response to environmental stresses. Numerous studies have revealed that *TCP* family genes are closely associated with stress tolerance mechanisms. For instance, in chickpea (*Cicer arietinum*), five *TCP* genes (*CaTCP3*, *CaTCP13*, *CaTCP15*, *CaTCP20*, and *CaTCP21*) containing MYB *cis*-acting elements were strongly induced under drought conditions, with similar results observed in other leguminous species [30]. In maize (*Zea mays*), drought stress significantly upregulated *ZmTCP42* and *ZmTCP32*, and the overexpression of *ZmTCP42* in *Arabidopsis* enhanced drought tolerance [31]. Similarly, in creeping bentgrass (*Agrostis stolonifera*), the overexpression of *Osa-miR319* suppressed its target genes (*AsPCF5*, *AsPCF6*, *AsPCF8*, and *AsTCP14*), enhancing tolerance to both drought stresses [32]. In rice (*Oryza sativa*), *OsTCP19* was found to modulate the ABA signaling pathway, enhancing tolerance to drought and high temperatures during both seedling and adult stages [33]. In apple (*Malus domestica*), *MdTCP46* was shown to negatively regulate the ABA signaling pathway and drought responses by interacting with *MdABI5* [34]. Similarly, in cotton (*Gossypium hirsutum*), several *GhTCP* genes were significantly upregulated under heat and drought stresses [35]. The heterologous expression of *MeTCP4* from cassava (*Manihot esculenta*) in *Arabidopsis* improved cold stress tolerance [36]. In *Liriodendron chinense*, *LcTCP* transcription factors, particularly *LcTCP1*, were responsive to drought and heat and cold stresses [37]. Together, these findings underscore the multifaceted roles of TCP transcription factors in plant growth, development, and stress tolerance. However, the specific expression patterns and functional mechanisms of *TCP* genes across different plant species require further in-depth exploration to comprehensively elucidate their contributions to plant adaptation and resilience.

*B. platyphylla*, a deciduous boreal tree native to the cold climates of Europe, Asia, and North America, is valued for its dense, fine-textured wood, which is extensively used in furniture, construction, and paper production [38]. Beyond its economic value, *B. platyphylla* plays a vital role in soil stabilization, water retention, and maintaining the balance of forest ecosystems [39]. This species is adapted to cold climates and exhibits seasonal resilience; however, climate warming poses a threat to its phenology, growth, and physiological processes. Rising temperatures, altered precipitation patterns, and increasingly frequent droughts are projected to severely disrupt boreal forest ecosystems [40,41]. Although *B. platyphylla*’s ecological adaptability and responses to climate change have been studied, research on the systematic identification and functional characterization of its TCP transcription factors under abiotic stress remains limited. The availability of the *B. platyphylla* genome sequence offers crucial genetic resources and data for comprehensive studies on the *TCP* gene family [42]. Therefore, exploring the *TCP* gene family in *B. platyphylla* is of substantial scientific importance.

This study identified 21 members of the *B. platyphylla TCP* gene family and performed a comprehensive analysis of their physicochemical properties, phylogenetic relationships, and gene structures. Using tissue-specific expression profiling and *cis*-acting element prediction, we investigated the regulatory responses of *BpTCP* genes to abiotic stresses such as drought, high temperatures, and cold stress. These findings deepen our understanding of the evolutionary history and functional characteristics of the *B. platyphylla TCP* gene family, offering a theoretical basis for genetic improvement to enhance its resilience to abiotic stresses.

## 2. Results

### 2.1. Identification and Physicochemical Properties of the BpTCP Gene Family

Two methods, the HMM search and bidirectional BLAST comparison, were used to identify *B. platyphylla* TCP family members. Functional domains of candidate proteins were analyzed with the SMART software (version V2.7.0.0), and proteins lacking conserved domains were excluded. The overlap of the results from the two methods identified 21 TCP proteins in *B. platyphylla*, which were renamed *BpTCP1* to *BpTCP21* according to their chromosomal locations. Detailed information regarding the physicochemical properties of the 21 BpTCP proteins is shown in Table 1. BpTCP proteins ranged in length from 123 amino acids (*BpTCP10*) to 799 amino acids (*BpTCP2*), with their molecular weights ranging from 14,007.67 kDa (*BpTCP10*) to 86,635.17 kDa (*BpTCP2*), showing considerable size variation among the TCP transcription factors. The theoretical isoelectric points (pI) ranged from 5.02 to 10.01, and all average hydrophilicity values were below 0, indicating strong hydrophilic properties. The average instability index of BpTCP proteins was 51.31, classifying most proteins as unstable, except for *BpTCP12*, *BpTCP17*, and *BpTCP18*. Subcellular localization indicated that most BpTCP proteins are located in the nucleus, highlighting their significance in the process of transcriptional regulation. However, *BpTCP7* and *BpTCP10* were localized to chloroplasts, and *BpTCP9* was localized to the cytoplasm. These findings indicate that BpTCP proteins mainly operate in the nucleus, with some members potentially involved in processes within chloroplasts or the cytoplasm.

### 2.2. Phylogenetic Analysis of the BpTCP Gene Family

**Figure 1:**
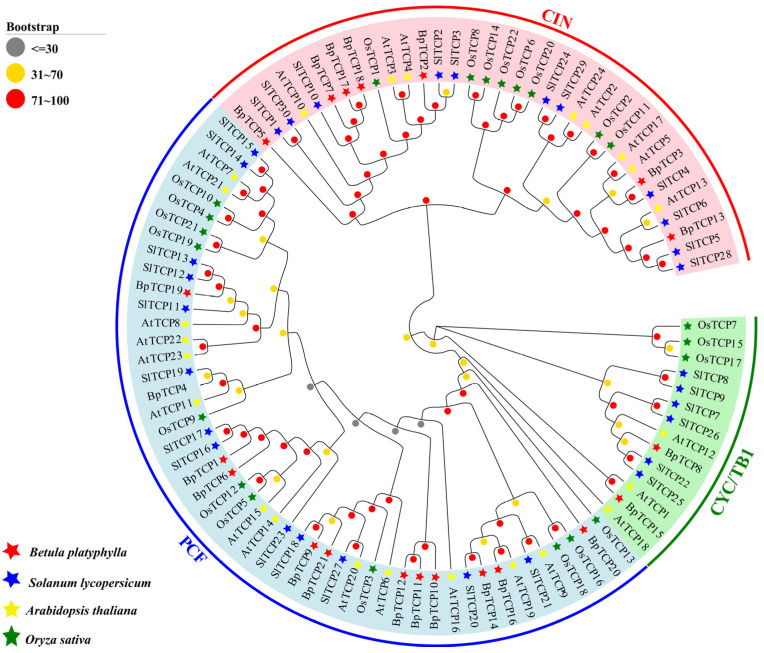
Phylogenetic analysis of *BpTCP* genes. Phylogenetic tree constructed based on TCP sequences of *B. platyphylla* (Bp), *Solanum lycopersicum* (Sl), *Arabidopsis thaliana* (At), and *Oryza sativa* (Os). Note: Pentagrams of different colors indicate different TCP classes. AtTCP18 is a functional member of CYC/TB1, but it appears in the PCF branch due to sequence-based phylogenetic clustering and low guidance support.

To investigate the evolutionary relationships of *TCP* genes in *B. platyphylla*, *Arabidopsis thaliana*, rice (*Oryza sativa*), and tomato (*Solanum lycopersicum*), a phylogenetic tree was generated using 97 TCP sequences from these four species (Figure 1, Table S1). The analysis revealed that *TCP* genes in *B. platyphylla*, *Arabidopsis*, rice, and tomato were clearly divided into two main classes: class I (PCF) and class II. Class II was further subdivided into two subfamilies: CIN and CYC/TB1. The distribution of *TCP* genes among these subfamilies differed between species. Among the 21 *BpTCP* genes identified in *B. platyphylla*, 12 belonged to the PCF subfamily, 7 to the CIN subfamily, and 2 to the CYC/TB1 subfamily. This variation in the number of *TCP* genes among subfamilies was consistent across species, with the PCF subfamily containing the most genes, followed by CIN, and with CYC/TB1 consistently containing the fewest.

### 2.3. Secondary and Tertiary Structure of BpTCP Proteins

**Figure 2:**
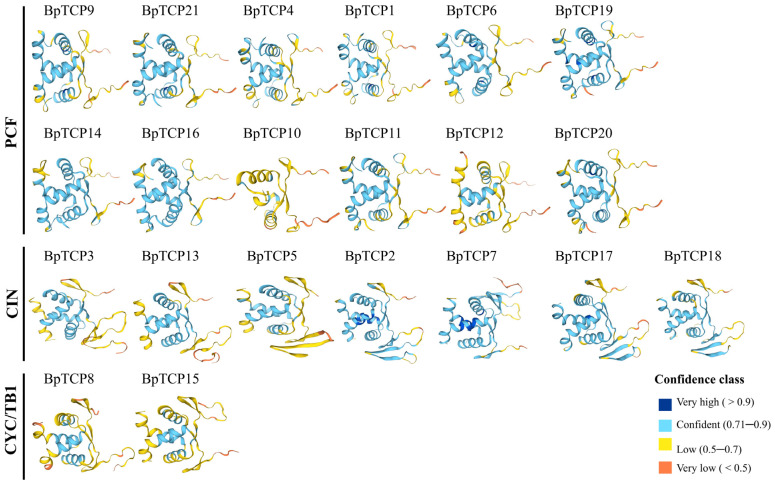
Analysis of the tertiary structure of the BpTCP proteins. The three subfamilies are labeled on the left, with the confidence levels of the proteins’ secondary structure represented by different colors, and the four levels are displayed in the lower-right corner.

SOPMA and SWISS-MODEL were employed to predict the secondary and tertiary structures of the *BpTCP* gene family proteins. Secondary structure analysis revealed that random coils and α-helices were the primary structural components (Figure S1). To gain deeper insights into the conserved structural domains of BpTCP proteins, we made predictive 3D structural models of all 21 BpTCP proteins using the SWISS-MODEL database. The quality of the predicted 3D structures was evaluated using the GMQE score, and the models were color-coded to represent varying confidence levels (Figure 2, Table S2). Significantly, the predicted models of the PCF subfamily exhibited higher confidence levels than the CIN and CYC/TB1 subfamilies did. The structural diversity observed among BpTCP proteins underscores the functional diversity within the *BpTCP* gene family. Comparative analysis based on protein database templates revealed symmetrical and highly similar structures among proteins within each subfamily. This suggests that the functions of the *BpTCP* gene family and its three subfamilies are largely conserved at the protein structure level, further highlighting their shared roles in various biological processes.

### 2.4. Multiple Sequence Comparison of BpTCP Proteins

**Figure 3:**
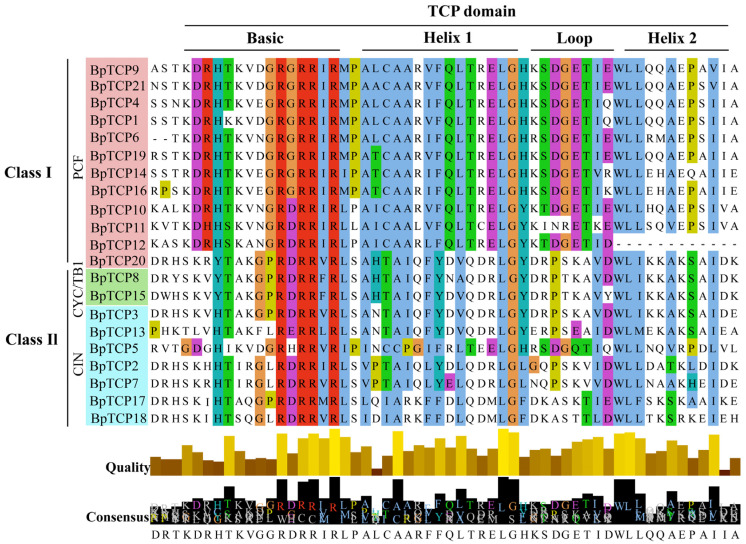
Multiple sequence alignment of TCP structural domains. Results of the sequence alignment of TCP structural domains in *B. platyphylla* TCP family members. The length of the rectangles below reflects the conservation level of amino acid positions, with longer rectangles indicating higher conservation; sequence indicators are displayed below.

We analyzed the sequence characteristics of 21 BpTCP proteins, focusing on their conserved structural domain sequences (Figure 3). Four conserved motifs were identified in BpTCP proteins: the basic region, helix I, loop, and helix II. The high degree of sequence alignment underscores the strong conservation of these motifs within each subfamily. Notably, class I proteins have four fewer amino acids in the basic region than those in class II but show higher overall conservation, clearly distinguishing the two subfamilies. Among the motifs, the basic region is the most conserved, followed by the helices, whereas the loop region displays the highest variability. Specific amino acid residues, including glycine (G) in the basic region, leucine (L) in the helices, and tryptophan (W), are fully conserved across all sequences, indicating their essential roles in maintaining protein structure and function. Remarkably, BpTCP12 proteins show notable amino acid deletions in both the loop and helix II regions, potentially driving functional divergence between class I and II proteins and affecting their regulatory roles and target gene expression. These findings indicate that different subfamilies may perform complementary biological functions, whereas members within the same subfamily may display functional redundancy.

### 2.5. Gene Structure and Conserved Motif Analysis

**Figure 4:**
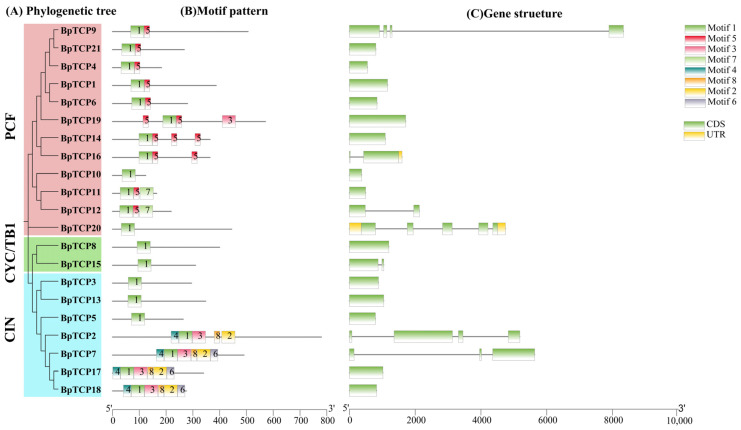
Analysis of conserved motifs and gene structures in the *BpTCP* gene family. (**A**) Phylogenetic classification of *BpTCP* genes, with the PCF subfamily represented by a red layer, the CYC/TB1 subfamily by dark green, and the CIN subfamily by light blue. (**B**) Identification of conserved motifs in BpTCP proteins, where distinct colored rectangles denote individual conserved regions. (**C**) Gene structure analysis of *BpTCP* genes, with green rectangles representing coding sequences (CDSs), yellow rectangles representing untranslated regions (UTRs), and black lines indicating introns.

Conserved motifs of BpTCP proteins were identified using the MEME tool, and the evolutionary tree was constructed and visualized with TBtools-II. The BpTCP protein family contains eight conserved motifs, labeled motifs 1 to 8 (Figure 4B). The composition and distribution of these motifs varied across BpTCP proteins. Motif 1 was present in all BpTCP proteins, while motif 5 was specific to the PCF subfamily but absent in BpTCP10 and BpTCP20. Phylogenetic analysis of the *BpTCP* gene family (Figure 4A) grouped the proteins into two subfamilies, class I (PCF) and class II (CYC/TB1 and CIN), aligning with previous findings. In the CYC/TB1 subfamily, motif 1 was the only conserved motif, whereas in the CIN subfamily, BpTCP7, BpTCP17, and BpTCP18 shared identical motif compositions, reflecting a high level of structural conservation. To gain a more profound understanding of the phylogenetic relationships and structural diversity of *BpTCP* genes, we analyzed the exon–intron structures of 21 *BpTCP* genes (Figure 4C). Fourteen genes were intronless, while seven contained between one and four introns, with BpTCP20 exhibiting the highest number (four introns). Additionally, untranslated regions (UTRs) were found in BpTCP16 and BpTCP20, highlighting structural variations within the gene family. These findings underscore the diversity of motifs and gene structures within the BpTCP family, potentially contributing to their functional specialization.

### 2.6. Chromosomal Localization of the BpTCP Gene Family

**Figure 5:**
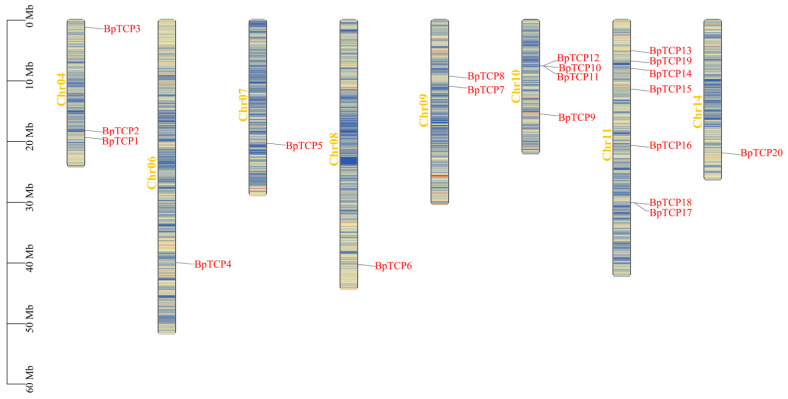
Chromosomal localization of the *BpTCP* gene. *B. platyphylla* has 14 chromosomes; scale bar is in Mb. Chr: chromosome.

The chromosomal localization analysis of *BpTCP* genes using genomic data from *B. platyphylla* (Figure 5) revealed a clear distribution pattern across eight chromosomes. Remarkably, the *BpTCP21* gene was not assigned to any chromosome. Additionally, no *BpTCP* genes were found on chromosomes 1, 2, 3, 5, 12, or 13. The distribution of *BpTCP* genes varied across chromosomes: chromosome 11 contained the most (seven genes), whereas chromosomes 6, 7, and 8 each had only one gene. Further analysis found no correlation between chromosome length and the number of *BpTCP* genes, indicating that gene distribution is independent of chromosome size.

### 2.7. Intraspecies Covariance Analysis

**Figure 6:**
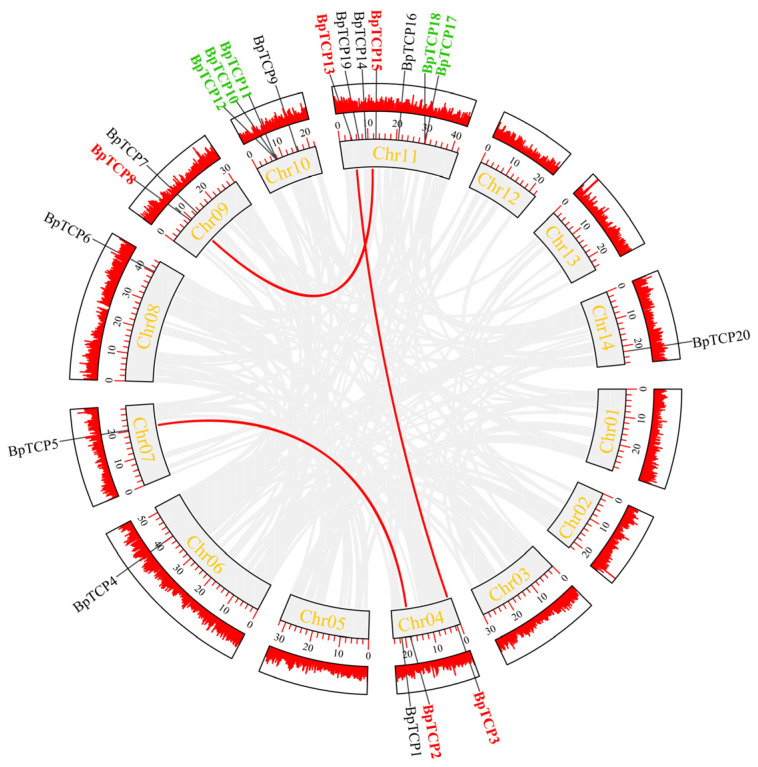
Intraspecies covariance analysis of *BpTCP* genes. The red line in the outer ring indicates the gene density per chromosome. Covariate genes are indicated by light gray lines. *BpTCP* gene pairs are labeled in red, while green indicates tandemly duplicated genes.

To further elucidate the evolutionary history of the *BpTCP* gene family in *B. platyphylla*, we conducted an in-depth analysis of gene duplication events (Figure 6), which revealed the occurrence of multiple gene duplication events within the *B. platyphylla* genome. On chromosome 11, a tandem duplication event was identified involving the gene pair *BpTCP17* and *BpTCP18*, while chromosome 10 displayed another tandem duplication involving BpTCP10, BpTCP11, and BpTCP12. Additionally, three pairs of segmentally duplicated genes were detected: BpTCP8 and BpTCP15; BpTCP2 and BpTCP5; and BpTCP3 and BpTCP13. These findings underscore the crucial role of gene duplication events in the evolution and expansion of the *BpTCP* gene family in *B. platyphylla*. Significantly, the quantity of segmentally duplicated gene pairs surpassed that of tandemly duplicated ones, suggesting that segmental duplications may have been a more significant driver of functional diversification and evolutionary complexity within the BpTCP family. Furthermore, the calculated Ka/Ks ratios, all of which were less than 1, indicate that these duplicated genes are under strong purifying selection, preserving their functional integrity and maintaining sequence conservation under evolutionary pressures (Table S3).

### 2.8. Interspecific Collinearity Analysis

**Figure 7:**
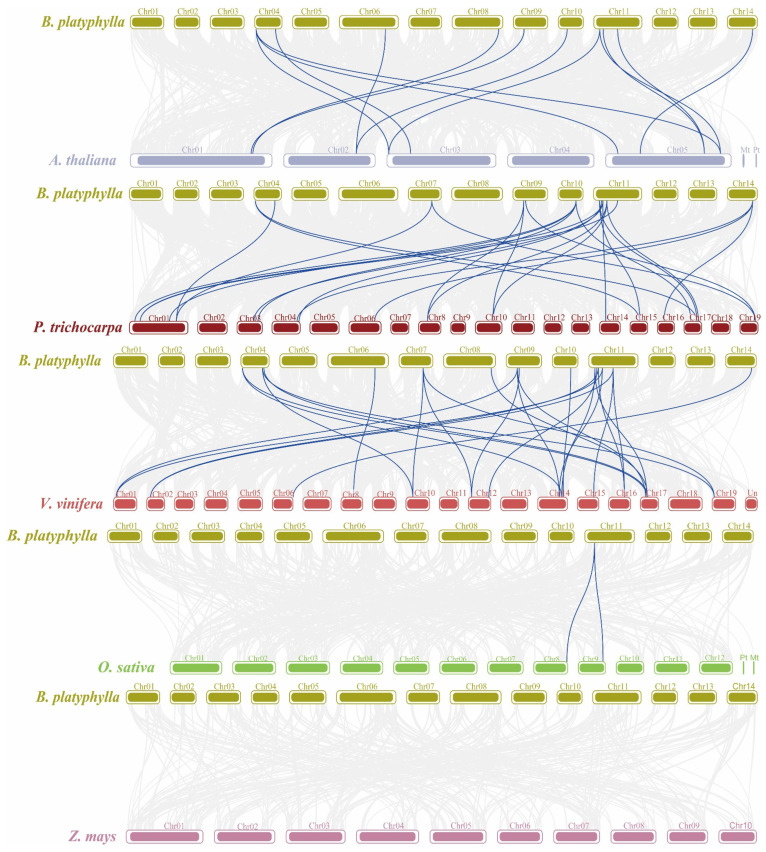
Covariance analysis of *BpTCP* genes with five representative plant species. Gray lines in the figure represent covariance regions between the genomes of *B. platyphylla* and other plants, while blue lines highlight covariant *TCP* gene pairs.

To further investigate the evolutionary trajectory of the *BpTCP* genome, we conducted a genomic synteny analysis with five representative species: three dicotyledonous plants (*Arabidopsis thaliana*, *Trichosanthes mauritiana*, and grapevine) and two monocotyledonous plants (rice and maize) (Figure 7). The analysis revealed varying degrees of genomic synteny between *B. platyphylla* and these species. A total of 14 homologous gene pairs were identified between *B. platyphylla* and *A. thaliana*, indicating a moderate level of synteny. Stronger synteny was observed with *T. mauritiana*, which shared 23 homologous gene pairs, while the highest degree of synteny was detected with grapevine, sharing 28 homologous gene pairs. By contrast, synteny with rice was limited, with only two homologous gene pairs, and no syntenic gene pairs were identified between *B. platyphylla* and maize. Notably, *B. platyphylla* exhibited significantly higher levels of synteny with dicotyledonous species than with monocotyledons. This finding suggests that *B. platyphylla* is more closely related to dicotyledonous plants such as grapevine, *Populus tremula*, and *A. thaliana* while being evolutionarily more distant from monocotyledons like rice and maize. These results further support the conclusion that dicotyledons share a closer evolutionary relationship with *B. platyphylla*.

### 2.9. Analysis of Promoter Cis-Acting Elements of the BpTCP Gene Family

**Figure 8:**
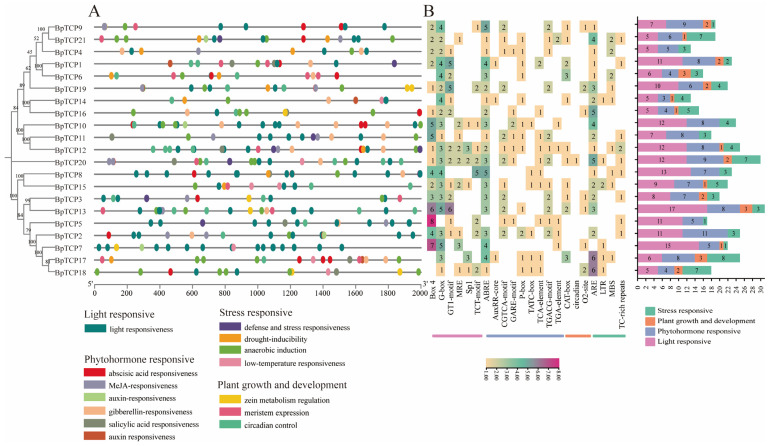
*Cis*-regulatory element (CRE) analysis in the promoter regions of *BpTCP* genes. (**A**) Distribution of various types of CREs in the promoter regions. Colored boxes represent different CRE categories, with some elements overlapping within the same regions. (**B**) Statistical representation of the number of CREs in the promoter regions of the *BpTCP* gene. The heatmap shows the abundance of each CRE, with white representing the absence of the corresponding element in the analyzed region.

To better understand the biological functions of *BpTCP* genes and their regulatory networks, we examined *cis*-regulatory elements within the 2000 bp upstream promoter regions of *BpTCP* genes. A total of 440 *cis*-regulatory elements (CREs) were identified and categorized into four groups: light response, stress response, hormone response, and growth and development response (Figure 8A, Table S4). Among them, light-responsive elements were the most abundant, accounting for 192 elements (43.6%), followed by hormone-responsive elements with 140 elements (31.8%). Growth- and development-related elements were the least abundant, with only 27 identified, indicating that the *BpTCP* gene family may play a relatively minor role in directly regulating plant growth and development. The widespread presence of light-responsive elements (Box 4), abscisic-acid-responsive elements (ABREs), anaerobic-inducible elements (AREs), drought-induced MYB-binding sites (MBSs), and low-temperature-responsive (LTR) elements suggests that the *BpTCP* gene family plays a key role in responding to diverse environmental stresses. Strikingly, the promoters of *BpTCP21*, *BpTCP4*, and *BpTCP6* each contained two MBS elements, indicating that these genes may act as key regulators in drought stress responses. Likewise, the promoter of *BpTCP15* contained two LTR elements, underscoring its potential role in low temperature stress responses. However, no CREs related to high temperature stress were identified. A total count of CREs across all *BpTCP* genes (Figure 8B, Table S5) revealed that *BpTCP13* and *BpTCP20* each contained over 30 CREs. In terms of adversity response elements, *BpTCP17* harbored eight stress-related elements, while *BpTCP18*, *BpTCP20*, and *BpTCP21* each contained seven, suggesting their critical roles in *B. platyphylla*’s responses to environmental stresses such as low temperature and drought. Phylogenetic tree analysis showed that class I genes (PCF subfamily) contained the highest number of stress-responsive elements at 47. This finding highlights the importance of class I genes in mediating *B. platyphylla*’s responses to adverse environmental conditions.

### 2.10. BpTCP Expression Profile in Different Tissues and During Drought

**Figure 9:**
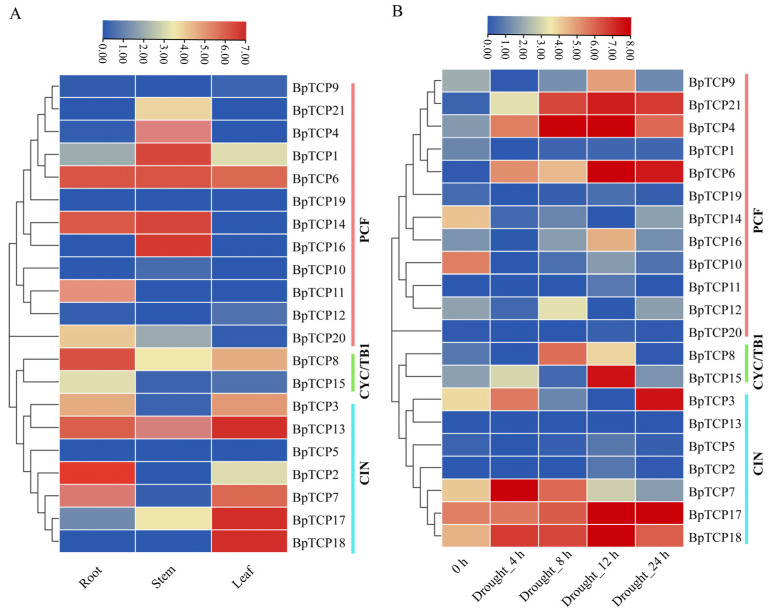
Analysis of *BpTCP* gene expression across different tissues and under drought stress. Expression profiles of *BpTCP* genes (**A**) in different tissues and (**B**) under drought stress. Note: The log2 values represent the relative expression levels of each *BpTCP* gene. The scale bar above each heatmap represents the relative expression levels, where red, yellow, and blue denote high, medium, and low expression levels, respectively. The legend on the right represents the phylogenetic subclasses, with light green, light blue, and dark red corresponding to the CYC/TB1, CIN, and PCF subclasses, respectively.

To better understand the roles of *BpTCP* genes in the growth and development of *B. platyphylla*, we analyzed their expression patterns across various tissues and organs (Figure 9A, Table S6). The analysis revealed substantial variations in the levels of gene expression among different tissues, with distinct expression patterns across subfamilies. Subfamily analysis indicated that CIN subfamily genes were highly expressed in the leaves of *B. platyphylla*, whereas PCF subfamily genes showed predominant expression in the stems. These findings suggest that *BpTCP* genes from different subfamilies have distinct functional roles in specific tissues. Specifically, *BpTCP2*, *BpTCP3*, *BpTCP14*, *BpTCP17*, *BpTCP16*, and *BpTCP18* were broadly upregulated in the roots, stems, and leaves, suggesting their involvement in common physiological processes across multiple tissues. Conversely, *BpTCP9*, *BpTCP21*, *BpTCP6*, *BpTCP19*, and *BpTCP13* were downregulated, implying that their functions may be suppressed under specific conditions or that they have limited roles in certain tissues. Additionally, several *BpTCP* genes exhibited distinct tissue-specific expression patterns. For instance, *BpTCP3*, *BpTCP7*, and *BpTCP18* were upregulated in the leaves, suggesting their roles in leaf development or responses to environmental stimuli. Similarly, *BpTCP2* and *BpTCP8* showed higher expression levels in the roots, suggesting their involvement in root growth, development, and water uptake. By contrast, *BpTCP1*, *BpTCP14*, and *BpTCP16* were predominantly expressed in the stems, indicating potential roles in stem development or environmental stress responses. These findings underscore the diverse and tissue-specific roles of *BpTCP* genes in *B. platyphylla*.

To investigate the response of *BpTCP* genes to drought stress, the relative expression levels of 21 *BpTCP* genes were assessed at five time points (0, 4, 8, 12, and 24 h) during drought treatment using qRT-PCR (Figure 9B, Table S7). The results revealed a distinct time-dependent and subfamily-specific expression pattern of *BpTCP* genes in response to drought stress. From a subfamily perspective, PCF subfamily genes exhibited a pronounced response to drought stress, with several genes showing significant expression changes. Specifically, *BpTCP19*, *BpTCP3*, *BpTCP13*, and *BpTCP5* exhibited downregulated expression under drought conditions, suggesting that these genes may be repressed during drought stress or lack direct involvement in drought-adaptive regulatory pathways. Conversely, CIN subfamily genes *BpTCP7* and *BpTCP18* were significantly upregulated as early as 4 h after drought treatment, indicating their roles as key regulators in the early drought stress response. As drought treatment progressed, the expression levels of *BpTCP21*, *BpTCP4*, *BpTCP6*, *BpTCP17*, and *BpTCP18* were markedly upregulated after 12 h, suggesting their primary involvement in the late drought stress response. Prominently, *BpTCP17* and *BpTCP18*, both from the CIN subfamily, exhibited strong and sustained expression responses throughout the treatment period. This suggests their potential roles in regulating long-term drought acclimation in *B. platyphylla*.

### 2.11. Expression Analysis of BpTCP Gene Under High- and Low-Temperature Treatments

**Figure 10:**
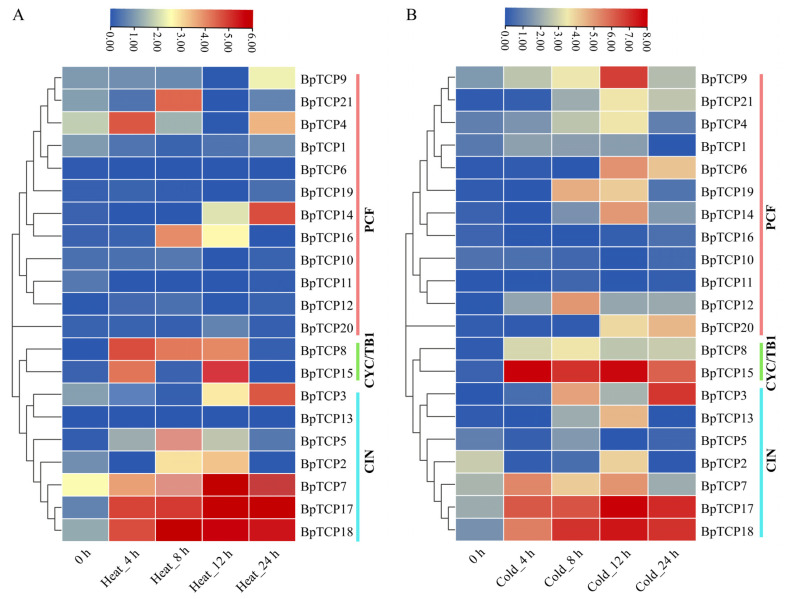
Expression profiles of *BpTCP* genes under high and low temperatures. Expression patterns of *BpTCP* genes under (**A**) high temperature and (**B**) cold stresses. Note: Log2 values indicate the relative expression levels of each *BpTCP* gene. The scale bars above the heatmaps indicate relative expression levels, with red, yellow, and blue corresponding to high, medium, and low expression levels, respectively. Phylogenetic subfamilies are shown on the right, with light green, light blue, and dark red indicating the CYC/TB1, CIN, and PCF subfamilies, respectively.

To investigate the expression patterns of *BpTCP* genes subjected to high temperature stress, the relative expression levels of 21 *BpTCP* genes were analyzed systematically using quantitative real-time PCR (qRT-PCR) at multiple time points (Figure 10A, Table S8). Under high temperature stress, CIN subfamily members such as *BpTCP7*, *BpTCP17*, and *BpTCP18* showed significant upregulation, emphasizing their unique stress-responsive roles. Among them, *BpTCP17* and *BpTCP18* maintained consistently high expression levels from 8 to 24 h, significantly higher than that of the control, suggesting their critical roles during prolonged high temperature stress. Time-course analysis showed that *BpTCP18* peaked at 8 h, indicating a rapid response to high temperature stress, while *BpTCP17* peaked at 12 h, suggesting its role in the middle and later phases of the stress response. Additionally, CYC/TB1 subfamily members, including *BpTCP8* and *BpTCP15*, displayed notable upregulation at specific time points (e.g., 8 and 12 h) under high temperature stress. Evidently, *BpTCP8* exhibited a temporally specific and auxiliary regulatory role, suggesting its involvement in the stress response of *B. platyphylla*. By contrast, most PCF subfamily genes were downregulated under high temperature stress, suggesting a reduced role for this subfamily in responding to such stress conditions. Instead, their primary functions are likely more related to plant growth and development processes than to environmental stress adaptation. For instance, although *BpTCP14* showed slight upregulation at specific time points (e.g., 12 h), its overall expression level remained low, supporting the notion that PCF subfamily genes play a minor role in high temperature stress responses.

To investigate the expression patterns of *BpTCP* genes under low temperature stress (Figure 10B, Table S9), we analyzed their transcriptional responses. *BpTCP17* and *BpTCP18* from the CIN subfamily showed significant upregulation under low temperature stress, highlighting their crucial roles in the abiotic stress response of *B. platyphylla*. Their expression levels were markedly high, as indicated by the dark red coloring, during the 8 and 12 h cold stress treatments. This expression pattern closely mirrors their response to high temperature stress, suggesting that these two genes may function as central regulators in temperature stress adaptation. Additionally, *BpTCP15* from the CYC/TB1 subfamily showed significant upregulation during the early (4 h) and middle (12 h) phases of low temperature stress. This time-specific expression indicates its potential involvement in signaling pathways or gene regulation during the early and intermediate phases of the cold stress response. By contrast, most PCF subfamily genes, such as *BpTCP1*, *BpTCP10*, and *BpTCP6*, showed low expression levels under low temperature stress, with limited or negligible responses. Only a few genes, such as *BpTCP14*, displayed weak upregulation at specific time points, suggesting that the PCF subfamily plays a relatively minor role in the response to low temperature stress.

### 2.12. Subcellular Localization of BpTCP Proteins

**Figure 11:**
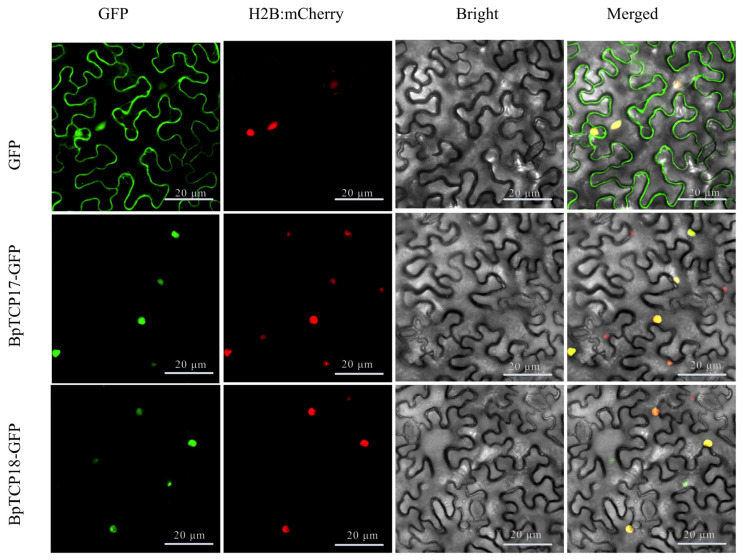
Subcellular localization of three representative TCP proteins. GFP is indicated as empty in the figure, and nuclear localization protein (H2B-mCherry) tagged using co-transformed mCherry was used to visualize the nucleus. Scale bar is 20 µm.

To identify the intracellular localization of BpTCP proteins, two cloned *BpTCP* genes (BpTCP17-GFP and BpTCP18-GFP) were inserted into the pCAMBIA1300-GFP vector under the control of the CaMV 35S promoter. The fusion constructs were then transformed into *Nicotiana benthamiana* epidermal cells and co-expressed with H2B-mCherry, a nuclear localization marker protein. Fluorescence microscopy showed that BpTCP17-GFP and BpTCP18-GFP fusion proteins emitted green fluorescence specifically in the nucleus (Figure 11). These findings confirm that BpTCP17-GFP and BpTCP18-GFP are nuclear-localized proteins, aligning with expectations and suggesting their potential role as transcription factors.

## 3. Discussion

The TCP family is a group of plant-specific transcription factors widely involved in diverse biological processes, including plant organ morphogenesis, responses to abiotic stresses, and hormone regulation. Identifying and characterizing the functions of *TCP* genes are crucial for understanding their roles in plant resistance to environmental stresses and can provide valuable genetic resources for improving stress tolerance in plants. With advancements in molecular biology and sequencing technologies, *TCP* gene families have been identified and reported in numerous plant species. For instance, 23 TCP members have been identified in orchids [43], 24 in *Arabidopsis thaliana* [44], 26 in rye (*Secale cereale* L.) [45], 50 in *Dendrobium chrysotoxum* [46], 30 in eggplant (*Solanum melongena*) [47], 49 in oats (*Avena sativa*) [48]. These quantitative differences across species may reflect variations in ecological adaptations, evolutionary histories, and mechanisms for responding to environmental stresses. Using homologous genes from *Arabidopsis thaliana*, all 21 *BpTCP* genes were classified into two major classes: class I (PCF) and class II (CIN and CYC/TB1). This classification aligns with findings in *Arabidopsis* [44], rice [49,50,51], and *Plumbago auriculata* [52], confirming the reliability of the evolutionary tree analysis. These results further emphasize the conservation and functional universality of *TCP* gene families across different plant species. Phylogenetic analysis indicates that the *BpTCP* genes are closely related to those from Arabidopsis thaliana and Oryza sativa. They cluster together in several branches, which suggests the presence of conserved evolutionary traits among these species. These commonalities likely reflect essential, conserved functions, particularly in gene regulatory networks involved in plant development and stress responses. However, the distinct segregation of several BpTCP genes into separate clusters, especially from Solanum lycopersicum, points to the potential for species-specific diversification, likely driven by ecological adaptations or distinct mechanisms for responding to environmental stress. Genes within the PCF and CYC/TB1 groups may represent functionally conserved clusters involved in critical processes such as organ development and abiotic stress resistance. The conservation of these clusters across diverse plant species implies that similar regulatory mechanisms have been preserved through evolution. This functional conservation suggests that *BpTCP* genes, akin to their homologs in Arabidopsis and rice, play key roles in plant growth, organ morphogenesis, and stress responses, processes shared by these plants due to their common evolutionary origins. Nevertheless, the distinct phylogenetic placement of some *BpTCP* genes hints at functional divergence, which may reflect specific adaptations to local environmental conditions or specialized regulatory roles in *B. platyphylla*, absent in Arabidopsis, rice, or tomato. For instance, certain *BpTCP* genes may have evolved to regulate stress response pathways unique to *B. platyphylla*, shaped by its particular ecological niche and environmental stresses.

Significant variation was observed in physicochemical properties among different BpTCP proteins. All BpTCP proteins were predicted to be hydrophilic, suggesting their strong hydration capacity and suitability for functioning in transcriptional regulation within the cell. This is consistent with previous findings in *Gossypium hirsutum* (cotton) [53] and sweet cherry [54]. Subcellular localization predictions indicated that most BpTCP proteins are localized in the nucleus, while a few are found in the cytoplasm or chloroplasts. It is worth noting that the subcellular localization of certain transcription factors is not strictly confined to the nucleus [55,56]. For example, *FvTCP7* in strawberries is localized in both the nucleus and cytoplasm [57]. Based on these findings, we selected *BpTCP* genes strongly associated with abiotic stress responses for subcellular localization experiments. The results showed that *BpTCP17* and *BpTCP18* proteins were predominantly localized in the nucleus, consistent with the typical localization of transcription factors in the nucleus [58,59,60]. These results are also consistent with studies in moso bamboo [61,62] and passion fruit [63], further supporting the conserved nuclear localization of TCP proteins in plants. However, BpTCP7, BpTCP10, and BpTCP9 showed non-nuclear localization, which could reflect alternative regulatory roles in the cytoplasm or chloroplasts, possibly related to post-translational modifications, protein–protein interactions, or interactions with other cellular compartments.

Gene structure and conserved motif analyses are essential for understanding gene function, evolutionary history, and classification. In this study, we analyzed the exon–intron structures of *BpTCP* genes and observed that members of the PCF subfamily often lack introns, a trend consistent with findings in the buckwheat *TCP* gene family [64]. Within each subfamily, the structural features of genes were highly similar, as seen in other plant species such as rye [45], further emphasizing the conservation of gene structure across different species. The arrangement and positions of exons and introns provides important insights into the evolutionary relationships of these genes. Conserved motif analysis revealed that all *BpTCP* genes retained motif 1, and the arrangement of motifs was closely associated with their evolutionary classification. This supports the notion that *TCP* genes underwent early divergence in plant evolution [65]. For instance, *BpTCP17* and *BpTCP18* from the CIN subfamily possessed the highest number of conserved motifs, with strong similarities in both their number and arrangement. This suggests that these two genes have preserved similar functional properties throughout their evolutionary history. Tertiary structure analysis further confirmed that proteins within the same subfamily shared highly similar structures, providing robust support for their functional studies. Chromosomal localization analysis showed that *BpTCP* gene family members were unevenly distributed across eight chromosomes in *B. platyphylla*. Interestingly, the *BpTCP21* gene was absent from the chromosomes, possibly due to gene loss events during evolution. Variations in the number of *TCP* gene family members across species may reflect genome duplication events during evolution. Our findings indicate that the expansion of the *BpTCP* gene family in *B. platyphylla* primarily resulted from segmental duplication rather than tandem duplication. This is consistent with the high degree of conservation of *TCP* gene families observed in species [66] such as citrus [67], poplar [68], and loblolly pine [69]. Ka/Ks ratio analysis revealed that all duplicated gene pairs had Ka/Ks ratios below 1, indicating that these genes have undergone purifying (negative) selection, preserving advantageous genetic variations during evolution. Furthermore, a covariance analysis of *BpTCP* genes across species showed no significant synteny with monocotyledonous plants but identified more covariant gene pairs with dicotyledonous species, such as *Arabidopsis thaliana*, *Hordeum vulgare*, and grapevine. This suggests that while *BpTCP* genes exhibit some level of conservation across plant species, they have also undergone distinct functional and expression divergence during evolution. These divergences may be closely linked to differences in plant morphology, life histories, and adaptations to varying environmental conditions.

Gene expression patterns are critical indicators of gene function [70]. Tissue-specific expression analysis showed substantial variation in the expression levels of 21 *BpTCP* genes across various tissues. *BpTCP6* and *BpTCP13* exhibited consistently high expression across all tissues, indicating their broad regulatory roles in the growth and development of *B. platyphylla*. Further analysis of their *cis*-acting elements identified three growth- and development-related elements in the promoter regions of both genes, emphasizing their critical roles in plant development. Phylogenetic analysis demonstrates that members of the CIN subfamily of TCP transcription factors inhibit cell proliferation in *Centaurea taurus*, with CIN deletion causing curled leaf margins [71]. Similarly, miR319 regulates CIN-like TCP transcription factors, resulting in an undulating or serrated leaf morphology [72]. In *B. platyphylla*, *BpTCP13*, *BpTCP17*, and *BpTCP18*—all members of the CIN subfamily—exhibited high expression in the leaves, indicating their key roles in regulating leaf size and shape. Similar research has also been conducted in Populus [73]. Additionally, *BpTCP1*, *BpTCP14*, and *BpTCP16*—members of the PCF subfamily—showed significant upregulation in the stems. Previous studies indicate that class I TCP transcription factors (members of the PCF subfamily) promote cell proliferation at the inflorescence branch tips, facilitating stem elongation [22]. These three *BpTCP* genes are hypothesized to play critical roles in regulating stem growth and development in *B. platyphylla*. *Cis*-acting elements related to environmental stress showed significant activity, particularly under drought and low-temperature conditions. Drought stress experiments revealed an increased expression of *BpTCP21*, *BpTCP4*, and *BpTCP6*, likely driven by drought-responsive *cis*-elements in their promoter regions. Similar findings have been reported in maize, where drought stress induced the expression of *ZmTCP42* and *ZmTCP32*, while the overexpression of *ZmTCP42* in Arabidopsis enhanced drought tolerance [31]. Conversely, the overexpression of *ZmTCP14* led to reduced drought tolerance [74]. Similarly, in *Allium* species, eight TCP genes, including *AsTCP17*, showed drought-inducible expression, and the heterologous overexpression of *AsTCP17* in *Arabidopsis* enhanced drought tolerance [75]. Crucially, in *B. platyphylla*, CIN subfamily members *BpTCP17* and *BpTCP18* were strongly upregulated under drought stress, whereas CYC/TB1 subfamily genes showed lower expression, underscoring the pivotal role of the CIN subfamily in drought stress responses. This observation aligns with findings in passion fruit [63]. Under low temperature stress, CIN subfamily genes *BpTCP7*, *BpTCP17*, and *BpTCP18* were significantly upregulated. Their promoter regions harbor low-temperature response *cis*-elements, further supporting their roles in cold stress adaptation. The role of CIN subfamily members in cold stress responses has been confirmed in various crops, including rice [49,76], sugarcane [77], Nanking chrysanthemum [78], and cassava [79]. Emphatically, *BpTCP15* exhibited high expression under low temperature stress, potentially linked to the presence of two low-temperature response elements (LTRs) in its promoter, suggesting its role in regulating the cold stress response via this pathway. Interestingly, although the typical heat stress response element (HSE) was absent in the BpTCP promoter regions, several genes still responded to high temperature stress. Expression analysis showed that CIN subfamily genes *BpTCP7*, *BpTCP17*, and *BpTCP18* were significantly upregulated under heat stress, suggesting their roles in high-temperature acclimatization in *B. platyphylla*. This aligns with findings in ginger (*Zingiber officinale*), where the CIN subfamily member *ZoTCP5* also exhibited strong upregulation under heat stress [80]. These findings highlight the conserved role of CIN subfamily TCP transcription factors in plant heat stress responses. Remarkably, further analysis revealed that *BpTCP17* and *BpTCP18* were significantly upregulated under high and low temperature and drought stresses. This indicates that these genes act as key regulators in *B. platyphylla*’s response to various environmental stresses. The upregulation of these genes likely improves plant physiology and metabolism, allowing *B. platyphylla* to better adapt to environmental changes through the regulation of growth and development.

## 4. Materials and Methods

### 4.1. Materials and Treatments

This study focuses on *B. platyphylla*, a species preserved in the laboratory of the Proteomics Research Center at the Strait Joint Research Institute of Fujian Agriculture and Forestry University. The *B. platyphylla* seedlings were cultivated in a greenhouse under controlled conditions, with a photoperiod of 16 h of light and 8 h of darkness, at a constant temperature of 25 °C. For tissue-specific expression analysis, 1-year-old seedlings with similar growth characteristics were selected to ensure uniformity. Root, stem, and leaf samples were collected separately for further analysis. The abiotic stress experiments consisted of both control and stress-treated groups, with each group containing three biological replicates of 12 seedlings per replicate. For the stress treatments, 3–5 mature leaves from the middle to the top of each seedling were randomly selected as samples. The experimental treatments involved drought and high and low temperature stresses. Drought stress was simulated by immersing the seedlings in a nutrient solution containing 10% polyethylene glycol (PEG), while control seedlings were placed in distilled water. High temperature stress was applied by placing the seedlings in a thermostatic incubator set at 40 °C, and low temperature stress was induced in an incubator set at 5 °C. Leaf samples from *B. platyphylla* seedlings were collected at various time points (0, 4, 8, 12, and 24 h) following the drought and high- and low-temperature treatments. Control samples were also collected at time 0. All samples were immediately frozen in liquid nitrogen and stored at −80 °C for subsequent analyses.

### 4.2. Genome-Wide Identification of TCP Gene Family in B. platyphylla

The TCP Pfam Hidden Markov Model (PF03634) was used to identify TCP structural domains in the genome-wide protein sequences of *B. platyphylla*. Candidate *TCP* genes were filtered with an E-value threshold of <0.001 to ensure high specificity. Known TCP protein sequences from *Arabidopsis thaliana* were simultaneously downloaded to construct a reference database. These sequences were compared against the *B. platyphylla* proteome database using BLASTp (https://blast.ncbi.nlm.nih.gov/Blast.cgi?PROGRAM=blastp, accessed on 10 April 2024), with the same E-value threshold (<0.001) applied to identify additional TCP candidates. Additionally, potential *TCP* genes were obtained from the NCBI database (https://www.ncbi.nlm.nih.gov/, accessed on 11 April 2024). Candidate *TCP* genes from the HMM search, BLAST comparison, and NCBI database were merged, and redundant sequences were eliminated. The non-redundant protein sequences were further analyzed for domain verification using three online tools: SMART (http://smart.embl.de/, accessed on 11 April 2024), Pfam (http://pfam.xfam.org/, accessed on 12 April 2024), and NCBI CDD (https://www.ncbi.nlm.nih.gov/cdd/, accessed on 12 April 2024). Proteins with TCP-specific structural domains were confirmed as members of the *TCP* gene family. The screened TCP protein sequences were analyzed for physicochemical properties, including isoelectric point (pI) and molecular weight (MW), using the ExPASy ProtParam online tool (http://web.expasy.org/protparam, accessed on 15 April 2024). Finally, the subcellular localization of *B. platyphylla* TCP family members was predicted using the WoLF PSORT online tool (https://www.genscript.com/wolf-psort.html, accessed on 15 April 2024).

### 4.3. BpTCP Protein Multiple Sequence Alignment and Evolutionary Relationship Analysis

BpTCP protein sequences were aligned using the CLUSTALW tool (https://www.genome.jp/tools-bin/clustalw, accessed on 3 May 2024) with default parameters [81], and the results were visualized using Jalview software (version 2.11.3.0) [82]. TCP protein sequences from potato (*Solanum tuberosum*), rice (*Oryza sativa*), and *Arabidopsis thaliana* were downloaded from the Plant Transcription Factor Database (http://planttfdb.cbi.pku.edu.cn/, accessed on 6 May 2024), along with *B. platyphylla* TCP protein sequences identified using the methods described earlier. Multiple sequence alignment of BpTCP protein sequences was performed using the MUSCLE algorithm in the MEGA11 (version 11.0.13) software [83] to ensure alignment accuracy. A phylogenetic tree of TCP proteins was constructed using the neighbor-joining (NJ) method, with a bootstrap value of 1000 iterations to ensure reliable evolutionary relationships. All other parameters were kept at their default settings. The completed phylogenetic tree was exported in “nwk” format and visualized using the iTOL online tool (https://itol.embl.de/itol.cgi, accessed on 10 May 2024) to provide a clear and detailed representation of the evolutionary relationships among BpTCP proteins.

### 4.4. Prediction of Secondary and Tertiary Structures of BpTCP Proteins

The secondary structure of BpTCP proteins was predicted using the SOPMA tool (https://npsa-prabi.ibcp.fr/cgi-bin/npsa_automat.pl?page=npsa_sopma.html, accessed on 14 May 2024) to analyze the structural composition of amino acid sequences. Homologous structures similar to BpTCP proteins were identified in the PDB database to provide a foundation for subsequent 3D structure prediction. The three-dimensional structures of BpTCP proteins were predicted using the Swiss-Model Interactive Tool (https://swissmodel.expasy.org/interactive/, accessed on 16 May 2024).

### 4.5. Gene Structure and Conserved Motif Analysis

The TCP protein sequences from *B. platyphylla* were analyzed for motif identification using the MEME online tool (http://meme-suite.org/tools/meme, accessed on 6 June 2024), with the number of motifs for CBLs set to 10 and the other parameters kept as the default. The motifs and conserved structural domains were visualized using the Gene Structure View tool (version 2.11.0).

### 4.6. Chromosome Localization and Covariate Covariance Analysis

Chromosomal localization and visualization of all *B. platyphylla* TCP gene members were conducted using the TBtools-II software (version 2.056) and the Gff annotation file of *B. platyphylla*. Intraspecific covariance analysis of *B. platyphylla* was conducted using MCScanX (https://github.com/wyp1125/MCScanX (accessed on 4 July 2024)) [84], with gene comparison performed via the native BlastP program and an E-value threshold of 1 × 10^−10^. MCScanX was subsequently used to generate covariance analysis files and classify the gene duplication types of the 21 *BpTCP* gene members. The coding sequence length (CDS), protein size, and Ka/Ks ratio were calculated using the Fasta Stats program and the Simple Ka/Ks Calculator (NG) in TBtools-II. The Ka/Ks ratio was used to assess gene differentiation tendencies following duplication events with the following criteria: Ka/Ks < 1 for purifying selection, Ka/Ks = 1 for neutral selection, and Ka/Ks > 1 for positive selection indicating accelerated evolution [85].

The results of intraspecific covariance analyses were visualized using the TBtools-II software to systematically present the covariance information of the *BpTCP* gene. For interspecific covariance analysis, genome files and Gff annotation files of Arabidopsis, rice (*Oryza sativa*), sorghum (*Sorghum bicolor*), and tomato (*Solanum lycopersicum*) were used. Subsequently, comparative cross-species analyses were conducted using the comparative genomics function of the TBtools-II software to map the genomes. The E-value threshold was set to 1 × 10^−10^, and default settings were applied to the other parameters.

### 4.7. Cis-Acting Element Analysis

The 2000 bp sequence upstream of the transcription start site of the *BpTCP* gene was extracted using the Seqkit v0.15 software [86] and submitted to the PlantCare website (https://bioinformatics.psb.ugent.be/webtools/plantcare/html/, accessed on 5 August 2024) for *cis*-acting regulatory element prediction. The prediction results were made de-redundant and quantified and visualized using TBtools-II.

### 4.8. RNA Extraction and qRT-PCR

RNA was isolated from control and stress-treated *B. platyphylla* leaf tissues using a HiPure Plant RNA Mini Kit (Magen). cDNA was synthesized using a PrimeScript RT Reagent Kit (Perfect Real Time, TaKaRa) (Nanjing Vazyme Biotech Co., Ltd., Nanjing, China). Quantitative real-time PCR (qRT-PCR) was used to analyze the expression profiles of 21 *BpTCP* genes across various tissues and under abiotic stresses, such as drought and high and low temperatures. Primers for qRT-PCR were designed using the Primer3 software (versionv4.1.0) (http://bioinfo.ut.ee/primer3-0.4.0/, accessed on 10 September 2024), with 18S rRNA serving as the internal reference gene [87]. All primers are listed in Table S10. The qRT-PCR conditions included pre-denaturation at 95 °C for 2 min, denaturation at 95 °C for 15 s, annealing at 60 °C for 30 s for 40 cycles, and a final extension at 72 °C for 30 s. Relative gene expression levels were calculated using the 2^−∆∆CT^ method [88], normalizing raw Ct values to the internal reference gene. Each experiment included three biological replicates and three technical replicates to ensure reliability. Statistical analysis of relative gene expression levels was performed using one-way analysis of variance (ANOVA), with a significance level of 5%. Gene expression profiles were visualized as heatmaps created with the HeatMap function in the TBtools-II software.

### 4.9. Subcellular Localization of BpTCP Proteins

*BpTCP17* and *BpTCP18* showed significant responses to multiple abiotic stresses based on expression analysis. To explore their functional roles, BpTCP17 and BpTCP18 were chosen for subcellular localization experiments. GFP tags were used to determine the subcellular localization of BpTCP17 and BpTCP18, following the method described by Xiaojin Huang et al. (2024) [89]. The primer information used for this analysis is listed in Table S10.

## 5. Conclusions

We identified 21 *BpTCP* genes in *Betula alba* and systematically analyzed their encoded proteins, focusing on physicochemical properties, evolutionary relationships, conserved motifs, gene structures, collinearity, and *cis*-acting elements. These genes show strong evolutionary conservation, highlighting their essential roles in plant development and environmental adaptation. Expression profiling demonstrated that *BpTCP* genes are crucial for *B. platyphylla* growth and development across diverse tissues. Promoter analysis revealed that *BpTCP* promoter regions contain abundant *cis*-acting elements linked to abiotic stress responses, including low-temperature responsiveness, drought tolerance, and defense mechanisms. Under abiotic stress conditions, CIN subfamily members showed substantial changes in gene expression, especially in response to drought and high and low temperature stresses. Significantly, *BpTCP17* and *BpTCP18* showed strong responses to these stressors, indicating their roles as key regulators in the adaptation of *B. platyphylla* to environmental challenges. Subcellular localization analyses confirmed that *BpTCP17* and *BpTCP18* are nuclear proteins, reinforcing their roles in stress responses via transcriptional regulation. Collectively, these findings provide valuable insights into the function of the *BpTCP* genes, which can be used in the future to validate the function of specific *BpTCP* genes (*BpTCP17* and *BpTCP18*) by overexpression or RNA interference techniques and to investigate their specific roles under different abiotic stress conditions.

## Figures and Tables

**Table 1:** Physicochemical characteristics of 21 *BpTCP* genes and their encoded proteins.

Gene ID	Protein ID	AA	MW	pI	II	AI	GRAVY	SL
BPChr04G00604	BpTCP1	386	40,843.18	6.87	66.17	56.71	−0.597	Nucleus
BPChr04G00664	BpTCP2	779	86,635.17	7.16	56.51	61.90	−0.760	Nucleus
BPChr04G05342	BpTCP3	294	33,563.17	6.45	47.85	62.07	−0.914	Nucleus
BPChr06G30802	BpTCP4	182	19,700.41	8.45	58.59	72.47	−0.358	Nucleus
BPChr07G18922	BpTCP5	263	30,545.66	5.12	69.49	47.91	−1.108	Nucleus
BPChr08G01309	BpTCP6	279	30,575.31	9.44	73.68	68.89	−0.594	Nucleus
BPChr09G29718	BpTCP7	490	54,167.47	8.20	45.87	68.57	−0.533	Chloroplast
BPChr09G29943	BpTCP8	399	44,122.23	8.15	50.89	63.36	−0.656	Nucleus
BPChr10G03521	BpTCP9	505	55,993.84	10.01	52.59	71.07	−0.655	Cytoplasm
BPChr10G17577	BpTCP10	123	14,007.67	9.97	48.03	77.80	−0.259	Chloroplast
BPChr10G17579	BpTCP11	164	18,128.67	5.77	46.78	73.72	−0.423	Nucleus
BPChr10G17585	BpTCP12	218	24,210.98	6.18	34.61	82.75	−0.139	Nucleus
BPChr11G05694	BpTCP13	347	38,726.77	6.85	48.58	62.97	−0.842	Nucleus
BPChr11G06894	BpTCP14	363	38,410.07	6.24	51.92	70.72	−0.389	Nucleus
BPChr11G07004	BpTCP15	309	35,088.50	7.16	44.20	64.11	−0.784	Nucleus
BPChr11G07178	BpTCP16	363	37,925.25	6.07	56.73	61.68	−0.525	Nucleus
BPChr11G17722	BpTCP17	339	37,499.35	9.22	37.38	63.66	−0.493	Nucleus
BPChr11G17777	BpTCP18	275	30,761.23	7.13	30.87	64.33	−0.697	Nucleus
BPChr11G18700	BpTCP19	570	60,307.68	6.91	64.31	52.86	−0.791	Nucleus
BPChr14G12311	BpTCP20	444	48,868.34	6.19	46.47	85.86	−0.280	Nucleus
BPunChr32695	BpTCP21	267	28,414.76	8.71	45.97	63.63	−0.498	Nucleus

Note: AA: number of amino acids; MW: molecular weight; pI: theoretical isoelectric point; II: instability index; AI: aliphatic index; GRAVY: grand average of hydropathicity; SL: subcellular localization.

## Data Availability

Data are contained within the article and the Supplementary Materials.

## Supplementary Materials

The following supporting information can be downloaded at https://www.mdpi.com/article/10.3390/plants14060880/s1.
