# Supplementary material for: Systematic Analysis of the Betula platyphylla TCP Gene Family and Its Expression Profile Identifies Potential Key Candidate Genes Involved in Abiotic Stress Responses
Source: Plants (Basel). 2025 Mar 11;14(6):880. doi: 10.3390/plants14060880 (PMC11944959; doi:10.3390/plants14060880)
Supplement: Supplementary file 1 [file plants-14-00880-s001.zip › Supplementary Figure and Table/Supplementary Figure S1.pdf]

Supplementary Figure S1. Prediction of secondary structural properties of BpTCP proteins

Protein	Proportion of secondary structure element (%)				Distribution of secendary structure element
	α-helix	Extended strand	β-turn	Random coil	
BpTCP1	11.92	4.92	0	83.16	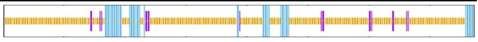
BpTCP2	14.89	7.96	0	77.15	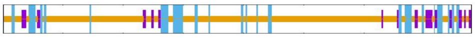
BpTCP3	12.24	5.44	0	82.31	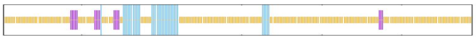
BpTCP4	15.38	4.95	0	79.67	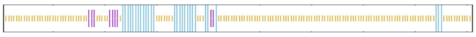
BpTCP5	17.11	6.46	0	76.43	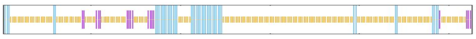
BpTCP6	15.05	2.87	0	82.08	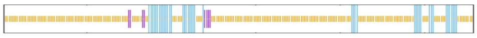
BpTCP7	12.86	10.00	0	7.14	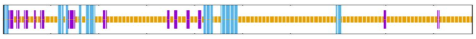
BpTCP8	15.79	3.51	0	80.70	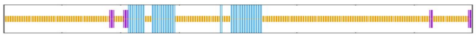
BpTCP9	23.37	11.29	0	65.35	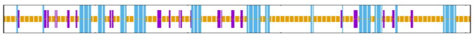
BpTCP10	25.20	11.38	0	63.41	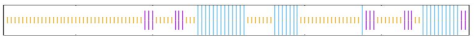
BpTCP11	30.49	4.27	0	65.24	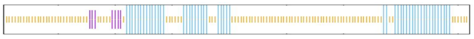
BpTCP12	23.96	6.42	0	70.18	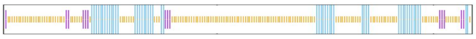
BpTCP13	11.24	5.19	0	83.57	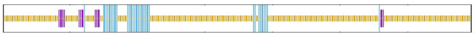
BpTCP14	11.02	5.51	0	83.47	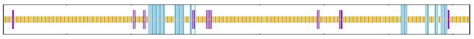
BpTCP15	23.30	4.21	0	72.49	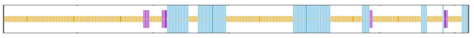
BpTCP16	11.29	4.68	0	84.02	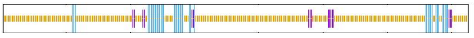
BpTCP17	10.62	8.26	0	81.12	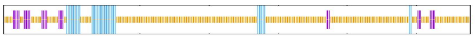
BpTCP18	11.64	5.82	0	82.55	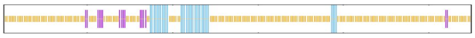
BpTCP19	7.54	9.65	0	82.81	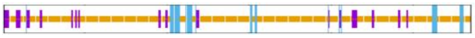
BpTCP20	23.65	19.59	0	56.76	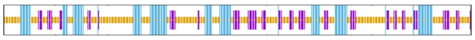
BpTCP21	12.36	4.49	0	83.15	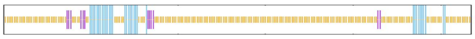
